# Recognising socio-cultural barriers while seeking early detection services for breast cancer: a study from a Universal Health Coverage setting in India

**DOI:** 10.1186/s12885-023-11359-3

**Published:** 2023-09-19

**Authors:** Riya Sawhney, Priyansh Nathani, Priti Patil, Prashant Bhandarkar, Deepa Kizhakke Veetil, Jubina Balan Venghateri, Nobhojit Roy, Anita Gadgil

**Affiliations:** 1 WHO Collaborating Centre for Research in Surgical Care Delivery in Low and Middle-Income Countries, Mumbai, India; 2 Department of Statistics, Bhabha Atomic Research Centre (BARC) Hospital, Mumbai, India; 3 Department of Minimal Access, General, Gastrointestinal and Bariatric Surgery, Manipal Hospitals, Delhi, India; 4 Department of Global Public Health, Karolinska Institute, 17177 Stockholm, Sweden; 5 The George Institute of Global Health, Delhi, India

**Keywords:** Breast cancer, Universal health coverage, Screening barriers, India

## Abstract

**Background:**

Breast cancer is the commonest cancer among women in India, yet the uptake of early detection programs is poor. This leads to late presentation, advanced stage at the time of diagnosis, and high mortality. Poor accessibility and affordability are the most commonly cited barriers to screening: we analyse socio-cultural factors influencing the uptake of early detection programmes in a Universal Health Coverage (UHC) setting in India, where geographical and financial barriers were mitigated.

**Methods:**

Two hundred seventy-two women engaging in an awareness-based early detection program were recruited by randomization as the participant (P) group. A further 272 women who did not participate in the early detection programme were recruited as non-participants (NP). None of the groups were previously screened for breast cancer. Interviews were conducted using a 19-point questionnaire, consisting of closed-ended questions regarding demographics and social, cultural, spiritual and trust-related barriers.

**Results:**

The overall awareness about breast cancer was high among both groups. None of the groups reported accessibility-related barriers. Participants were more educated (58.09% vs 47.43%, *p* = 0.02) and belonged to nuclear families (83.59% vs 76.75%, *p* = 0.05). Although they reported more fear of isolation due to stigma (25% vs 14%, *p* = 0.001), they had greater knowledge about breast cancer and trust in the health system compared to non-participants.

**Conclusions:**

The major socio-cultural barriers identified were joint family setups, lower education and awareness, and lack of trust in healthcare professionals. As more countries progress towards UHC, recognising socio-cultural barriers to seeking breast health services is essential in order to formulate context-specific solutions to increase the uptake of early detection and screening services.

**Supplementary Information:**

The online version contains supplementary material available at 10.1186/s12885-023-11359-3.

## Background

Breast cancer (BC) is the leading cancer amongst women in India [1]. Although the incidence of BC in India is only one-fourth that of high-income countries (HICs), the mortality rate is similar, at 13.3 per 100,000 in India compared to 12.9 per 100,000 in HICs [1]. One of the major causes of this disproportionately high mortality is the advanced stage of cancer at the time of presentation [2]. Breast cancers in the Indian subcontinent also occur at younger ages compared to HICs [3]. The World Health Organization (WHO) and Breast Health Global Initiative have recommended breast cancer awareness and clinical breast examination-based early detection programs for reducing the high mortality from breast cancer in Low- and Middle-Income countries (LMICs) such as India [4]. These programs aim to create breast cancer awareness at the community level and encourage people to attend clinical breast examinations (CBE). Most LMICs recruit frontline healthcare workers for these examinations at primary healthcare centres (PHCs), or other centres near womens’ places of residence [5]. The uptake of these screening and early detection programmes in India and other LMICs is low, as demonstrated by facility- and community-based studies [6, 7].

In 2010, the Government of India, through its National Health Mission, first launched a comprehensive non-communicable disease control program [8]. However, The National Family Health Survey, which is the largest household survey in India, documented that less than two percent of women have been screened for breast cancer in India [9]. Studies from LMICs attribute poor access to healthcare facilities, poor socio-economic conditions, as well as lack of awareness and education, and socio-cultural barriers as the main reasons behind limited uptake of screening services [10, 11]. Furthermore, stigma is also considered a key social determinant of health, and increasingly recognized as a significant psycho-social health barrier [12]. There is an increasing focus on exploring and addressing cancer-related stigma in India [13, 14]. Additionally, household factors such as family set-ups and autonomy to make health-related choices and decisions can also influence health-seeking behaviours, especially among women [15].

Universal Health Coverage (UHC) provides financial protection to patients and their families from out-of-pocket expenditure where healthcare provision is concerned [16]. Indian populations do not benefit from UHC and this contributes to delays in seeking, reaching, and completing cancer care [2]. Women in India, like in other LMICs [17], grapple with issues related to poor access and affordability of healthcare. This study was conducted in an Indian cohort that is covered by UHC, which essentially eliminated the affordability and accessibility issues, bringing focus to the socio-cultural barriers. As India and many other countries progress toward implementing UHC on a wider scale, it is informative and insightful to have a more nuanced evaluation of social and cultural barriers to the uptake of an early detection programme in a UHC setting, where other barriers have been largely mitigated. This study therefore aimed to evaluate barriers to seeking early detection services for breast cancer in an urban population covered by a UHC scheme.

## Methods

### Study setting

This study was conducted in an urban community cohort in Mumbai, India. This urban community of 100,000 people is covered under an employees’ contributory health service scheme. Under this scheme, healthcare services are provided at a minimum flat contribution of one percent of the basic pay of the employee. Most families in this cohort were of middle socio-economic status and earned a fixed salary in the Department of Atomic Energy employee cohort. The health system is structured in the form of a two-tiered system within the eastern suburbs of Mumbai, India. It consists of 14 PHCs spread across the city. The PHCs refer patients to a central referral hospital when needed. The 14 PHCs served a population of 100,000, and 4 of these 14 centres were transformed into breast clinics for this study [18]. In June 2013, an awareness-based early detection program for breast cancer (primarily using CBE and ultrasound) was implemented through a dedicated network of breast clinics across these PHCs, in collaboration with the International Agency for Research in Cancer (IARC). Approximately 22,500 women in the age group of 30–69 years were eligible for the early cancer detection program as per the Government of India Ministry of Health guidelines for screening in India [8].

Gadgil et al. have published further details about this healthcare system, cohort, and organisation, in a separate manuscript dedicated to methodology of establishing an early detection program [18]. The women in this cohort had not received any screening or early detection for breast cancer prior to the implementation of this program. They were given information on breast cancer awareness, signs and symptoms, and mail invites for early detection, and symptomatic as well as asymptomatic women were encouraged to report to clinics nearest to their homes. Some symptomatic women who participated were under the age of 30; however, as service providers, we could not deny clinical breast examinations to these patients, so they were also included in the study. None of the women were given any compensation or incentives for participation in the program. All participants resided in the catchment area around the PHCs and could access the screening activities without any cost [18].

### Study design

This prospective nested case–control study, conducted from June 2015 to June 2017, assessed the breast health-seeking behaviour of women who did not participate in the early cancer detection program, hereafter referred to as non-participants (NP). These non-participant women were compared to women who availed the program, henceforth referred to as participants (P), or the control group.

For the study, 80% power, 95% confidence intervals, and 12% participation rate in the program were attributed. Allowing for 10% attrition, *n* = 272 in each group was reached as the sample size.

$$n= \frac{{Z}^{2} p (1-p)}{{e}^{2}}$$

where,

Z = Confidence level at 95% (Standard value of 1.96)

p = Estimated prevalence or proportions

e = range of CI (5% or 10%)

The study was approved by the Ethics Committee of Bhabha Atomic Research Centre Hospital before starting the recruitment process (IRB approval number: BHMEC/NP/11/2016). All methods were carried out in accordance with relevant guidelines and regulations.

### Diagnosis and treatment protocol

All women who were detected with abnormalities on clinical breast examinations were referred to the hospital situated in the same residential area for further investigation and treatment. Diagnostic mammography and cytology were carried out as a part of triple assessment, but not as a screening modality. CBE remained the sole screening modality for all women [18].

### Recruitment and data collection

We recruited women who visited PHCs for complaints other than breast-related symptoms and those who were accompanying their relatives to health centres as ‘non-participants’ (NP). All women who had participated in the early detection program were identified for the control group (P). We generated a randomised list of unique identification numbers (UIDs) from the list of participants, and recruited these women for the study. Recruitment was conducted in keeping with a 1:1 allocation ratio between participants and non-participants. We contacted the recruited individuals over the phone and arranged a convenient time and date for in-person interviews. Informed consent was obtained from all women during their hospital visit prior to interview. When recruited women were illiterate, informed consent was obtained from their respective legal guardians.

Investigators were medical professionals with graduate and above level qualifications, with an experience of greater than 10 years at the community healthcare level. They attended a two-day training programme at the Department of Surgery at the central referral hospital where the early detection program was being implemented.

Trained investigators then conducted in-person, face-to-face interviews with all participants. If an individual in the P or NP groups refused to be interviewed or could not be contacted, the next individual in the initial randomised list was contacted, and so on.

Recruitment and interviews were completed over two rounds. In the first round of six months, the target sample size number (*n* = 272) was not reached due to low response rates in both P and NP groups. More UIDs were generated and randomised again to recruit participants, and non-participants were recruited from PHCs, as explained above. This additional set allowed us to reach the target sample size in the second round.

The investigators designed a questionnaire available in an additional file (see Additional file 1) addressing the well-documented barriers to breast health seeking in the literature discussed earlier. The questionnaire comprised 19 questions and was adapted from existing literature and questionnaires on the known social, cultural, and perceived risk-related barriers to breast health-seeking [19, 20]. Social desirability bias was controlled by appropriate framing of the questions included in the questionnaire. The questions were framed with both positive and negative wording to reduce response bias. The respondents were anonymized during analysis and confidentiality was maintained to further reduce social desirability bias.

The questionnaire contained demographic variables and included age, education, partner status, employment status, and family type (joint or nuclear). Joint family set ups refer to two or more married couples of a single generation, or three or more married couples from multiple generations living in the same household. On the other hand, nuclear families refer to a single married couple living with or without their unmarried children [21]. Questions related to the utilisation of and trust towards the healthcare system in general, and the ongoing early detection program specifically, were also included. The investigators conducted interviews in English, and in the national language Hindi, where participants were not fluent in English.

### Statistical analysis

SPSS Version 24.0 (SPSS Inc., IBM, Chicago, US) and Microsoft Office Excel 2013 for Windows were used to perform statistical analysis. Socio-demographic characteristics of women were represented across both groups. Responses were presented in absolute numbers as well as percentages. Chi-square test of significance was used, and *p* < 0.05 was set as the significance threshold.

## Results

**Fig. 1:**
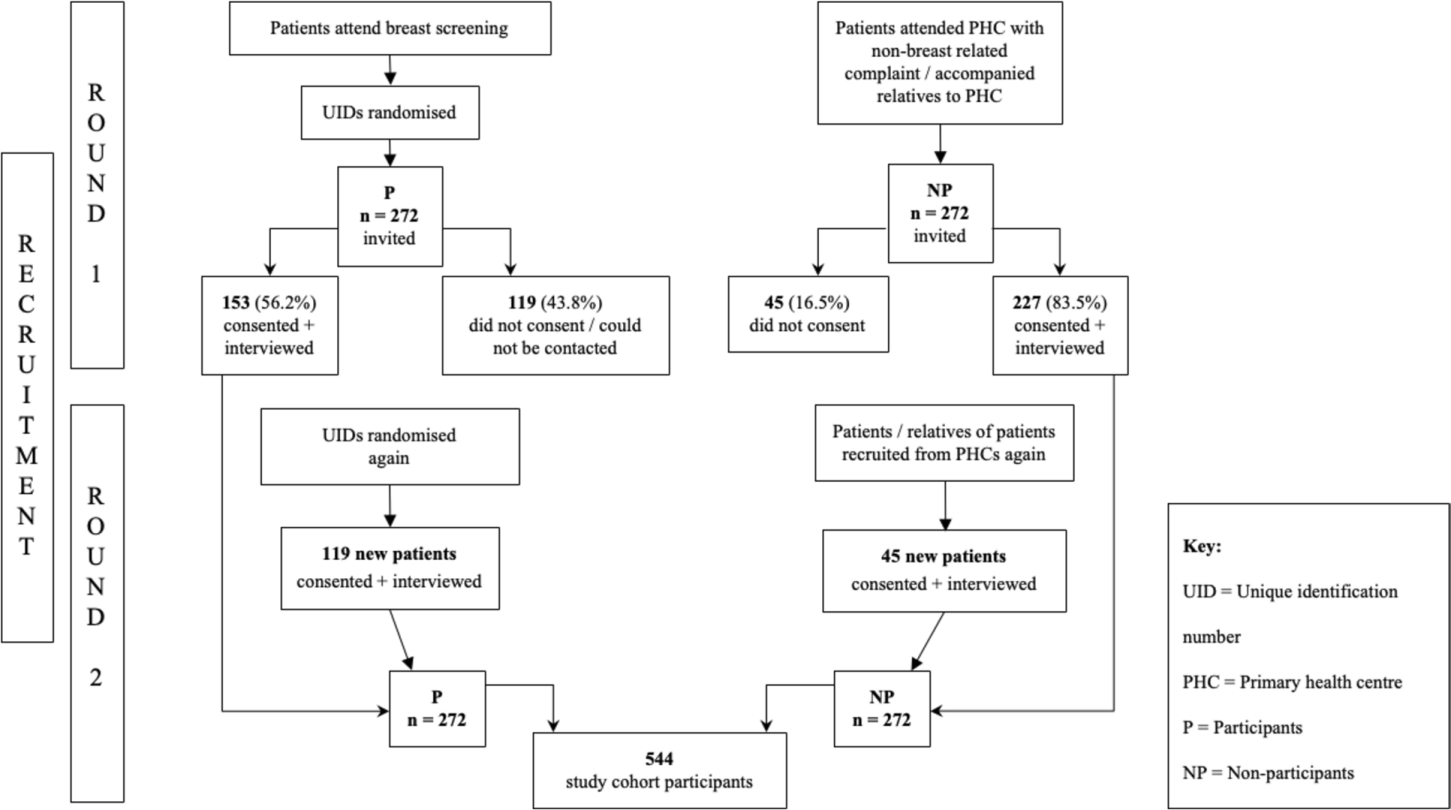
Recruitment algorithm

The total number of women recruited was 544: 272 participants and 272 non-participants. Figure 1 summarises the recruitment algorithm. The ages of women in this study ranged from 19 to 77 years. The mean age was 48.03 ± 10.37 years.

Table 1 shows the comparison of characteristics of women between NP and P groups. Participants were significantly more educated than non-participants, with a higher percentage of women receiving education up to and above the level of a graduate degree (58.09% vs 47.43% p = 0.02). Women who belonged to a higher-risk group of developing breast cancer, with a family history of breast and related cancers, participated more in the programme compared to women with average risk (19.03% vs 9.56% *p* < 0.01). Additionally, women from nuclear families had significantly more participation in the early detection programme (83.59% vs 76.75%, *p* = 0.05). Both groups were comparable in age, partner status, and occupation status.

**Table 1:** Characteristics of participants and non-participants

Narration	Participants	Non-Participants	Total	Missing (%)	*p*-value
**Age in Years** **(272 vs 272)**					
< 30	4 (1.47%)	3 (1.1%)	7	0	0.38
30–39	49 (18.01%)	56 (20.59%)	105		
40–49	108 (39.71%)	92 (33.82%)	200		
50–59	76 (27.94%)	95 (34.93%)	171		
60–69	24 (8.82%)	18 (6.62%)	42		
> 69	11 (4.04%)	8 (2.94%)	19		
**Education** **(272 vs 272)**					
Illiterate	15 (5.51%)	27 (9.93%)	42	0	0.02
School	99 (36.4%)	116 (42.65%)	215		
Graduate & Above	158 (58.09%)	129 (47.43%)	287		
**Partner Status** **(272 vs 272)**					
Partnered	256 (94.12%)	252 (92.65%)	508	0	0.49
Non-partnered	16 (5.88%)	20 (7.35%)	36		
**Occupation** **(264 vs 272)**					
Working	75 (28.41%)	66 (24.26%)	141	8 (0.01%)	0.28
Non-working	189 (71.59%)	206 (75.74%)	395		
**Menstrual Status (259 vs 265)**					
Post-menopausal	120 (46.33%)	107 (40.38%)	227	20 (3.68%)	0.17
Pre-menopausal	139 (53.67%)	158 (59.62%)	297		
**Family History of Breast and Related Cancer** **(268 vs 272)**					
Yes	51 (19.03%)	26 (9.56%)	77	4 (0.01%)	0.00
No	217 (80.97%)	246 (90.44%)	463		
**Family Type (262 vs 271)**					
Joint	43 (16.41%)	63 (23.25%)	106	11 (0.02%)	0.05
Nuclear	219 (83.59%)	208 (76.75%)	427		

### Awareness about breast cancer

Women in both groups had high breast cancer awareness, but awareness was higher among participants (98% vs 94%, *p* = 0.009). Awareness regarding breast lumps as a symptom of BC was comparable across groups. However, participant women were significantly more aware that cancer could also present as blood-stained nipple discharge (78% vs 69%, *p* = 0.048), skin changes over the breast (77% vs 54%, *p* < 0.01) or a lump in the armpit (90% vs 78%, *p* = 0.01). Furthermore, those who participated in the early detection program were significantly more aware of the need for breast examination in asymptomatic women (97% vs 83%, *p* < 0.01).

### Social and cultural barriers

Both groups of women agreed that their health was important and that their family and/or spouses would support their attendance at breast clinics for routine check-ups. Significantly more women in the NP group felt they did not have time to attend routine check-ups (15% vs 28%, *p* < 0.001).

A significant cultural barrier among participants was the social stigma of being labelled a “cancer patient” in the eyes of friends and neighbours, if seen attending breast clinics (25% vs 14%, *p* = 0.001). Fatalistic attitudes and spiritual beliefs regarding BC were similar in participants and non-participants, with no significant differences noted. None of the women perceived the ‘distance to clinics’ to be prohibitive or ‘accessibility’ as a barrier to attending the early detection program.

Though there was no perceived risk of overdiagnosis in either of the groups, participants were more worried about losing the entire breast if detected with a lump or cancer (48% vs 39%, *p* = 0.044). There was a statistically significant difference in patients’ trust in the doctors’ ability to treat BC if found, between participants and non-participants (100% vs 48%, *p* < 0.001). Furthermore, there was a significant difference in the reported rudeness and indifference of staff experienced by non-participants (0% vs 47%, *p* < 0.001). Table 2 describes the socio-cultural barriers to breast health seeking in both groups.

**Table 2:** Barriers as reported by participant (P) and non-participant (NP) groups

Question	(P vs NP)	Participants (P)		Non-Participants (NP)		Significance Testing
		Yes (%)	No (%)	Yes (%)	No (%)	*p*-value
**Social and Cultural Barriers**						
Do you feel it is necessary to have a breast check-up even in the absence of symptoms?	(270 vs 271)	262 (97)	8 (3)	224 (83)	47 (17)	0.000
Do you feel it is more important to look after the health of your family members than your own?	(270 vs 272)	124 (46)	146 (54)	116 (43)	156 (57)	0.442
Do you feel your family members or spouse would accompany you/encourage you for routine check-ups?	(267 vs 267)	242 (91)	25 (9)	241 (90)	26 (10)	0.883
Do you feel you would not have time to attend breast check-ups?	(267 vs 268)	39 (15)	228 (85)	74 (28)	194 (72)	0.000
Are you afraid that your neighbours or friends may see you as a “cancer patient” if you attend breast check-ups?	(268 vs 258)	66 (25)	202 (75)	35 (14)	223 (86)	0.001
**Geographical Barriers**						
Are the dispensary and breast examination service located prohibitively far away from your residence?	(269 vs 268)	40 (15)	229 (85)	30 (11)	238 (89)	0.206
**Perceived Risk**						
Do you fear that once you show a doctor, they will find some problem and prescribe further (perhaps unnecessary) treatment?	(269 vs 268)	105 (39)	164 (61)	94 (35)	174 (65)	0.342
Do you feel if a lump/cancer is detected you will lose your breast?	(241 vs 236)	116 (48)	125 (52)	92 (39)	144 (61)	0.044
**Fatalism and Spirituality**						
Do you think breast cancer is curable?	(239 vs 235)	209 (87)	30 (13)	208 (89)	27 (11)	0.722
Do you think breast cancer is a sin/punishment for sin from God?	(265 vs 265)	12 (5)	253 (95)	20 (8)	245 (92)	0.145
Do you think alternative medicines/spiritual healing is better than a doctor's medication or surgery?	(256 vs 249)	21 (8)	235 (92)	16 (6)	233 (94)	0.443
**Healthcare Provision Related Issues**						
Do you think your doctors and hospital can treat you if you are detected with breast cancer?	(272 vs 264)	271 (100)	1 (0)	128 (48)	136 (52)	0.000
Have you ever felt there was lack of privacy at the centre?	(270 vs 263)	2 (1)	268 (99)	6 (2)	257 (98)	0.144
Were the staff rude or indifferent to your desire to get routine check-ups?	(270 vs 264)	0 (0)	270 (100)	125 (47)	139 (53)	0.000

## Discussion

This study revealed that participants had higher levels of education, were more aware of the signs and symptoms of breast cancer, and the need for participation in early detection programmes even in the absence of symptoms. Non-participants belonged to joint families, and stated lack of time as a reason for not attending the programme. Despite higher education and awareness, participants feared social stigma and ‘losing the entire breast if detected with cancer’ but had significantly more faith in the health system, in that their doctors would be able to treat them if diagnosed with BC.

Education and awareness have repeatedly been cited as facilitators of early health-seeking behaviour [22–25]. Similarly, in our comparative study, participants had a significantly higher level of education and demonstrated greater awareness (as can be expected following participation in an awareness-based early detection programme such as ours). However, it is important to note here that although non-participants had lower knowledge compared to participants, their overall knowledge of breast cancer was still quite high – this is likely due to the fact that overall education levels were also quite high compared to other LMIC populations. Vieira et al. [22] examined the healthcare system related barriers in a systematic review in Brazil. They identified that people with less education had difficulty navigating and accessing the healthcare system for diagnosis and treatment. Low awareness of the presenting signs and symptoms of cancer is one of the major factors contributing to delayed presentation [23] and poor health-seeking behaviour among women [24]. Greater knowledge about the disease condition was noted to be one of the best predictors of adherence to recommended breast screening programmes in a study by Charkazi et al. [25]. In its early stages, cancer either has minimal symptoms or can be entirely asymptomatic. Thus, early detection is of utmost importance for better survival. However, without awareness of the disease, seeking breast examination becomes a low priority and is ignored, leading to very late-stage diagnoses.

A recent systematic review on barriers to breast and cervical cancer screening [10] found that in addition to common accessibility and affordability barriers, awareness and anxiety about disease and diagnosis were the most frequent socio-cultural barriers to participation in screening. Our UHC-based study largely mitigated the geographical and financial barriers. In this context, awareness and higher education in the population can be important determinants of participation in early detection and screening programs. Both formal education and disease awareness are consistently associated with better participation [10, 26], as is echoed in the findings of our study.

Women are often expected to be primary caregivers for dependent children and the elderly due to set societal gender roles, especially in the Indian subcontinent. This could lead to self-neglect and not viewing their own health as a priority. Studies from Brazil and Chile have highlighted that self-neglect as a result of limited time, has led to a decrease in women performing breast self-examinations [27]. Furthermore, the time taken to attend clinics and undergo investigative procedures has also been cited as a barrier to regular breast check-ups [28]. Families with dependent children also lead to decreased time for primary caregivers for health-seeking activities. These findings were similar to the results of our study, wherein we found that non-participants largely belonged to joint families and stated lack of time as a barrier to attending the early detection programme. Women from nuclear families had significantly greater participation, suggesting that joint family setups could compromise women’s autonomy to take decisions about resource expenditures and health-related decisions. Mahalakshmi et al. [7] similarly documented that family responsibilities were a deterrent to women’s participation in screening. Koirala [29] found that joint families were associated with fewer cancer diagnoses: the author elucidated that living in a joint family lowers the financial resources available to each member of the family to participate in health-seeking activities that would allow them to get diagnosed. In our cohort, where financial resources did not limit participation due to UHC, it is highly likely that lower autonomy in a joint family was the most influential factor. In a patriarchal society such as India, systemic gender inequities mean that women disproportionately take on household responsibilities, which in turn prevents them from having the autonomy to make health-related decisions. While there is intergenerational help and deep family solidarity in a joint family set-up, there is also a loss of privacy and autonomy. Traditional male authoritarian leadership shapes hierarchical relationships in a joint family in India: male sons must defer to their fathers, and women, especially daughters-in-law, are considered subordinate, lacking decision-making ability [30]. Recent work by the author team assessing the effect of women empowerment indices on screening uptake also showed similar findings: women who lack autonomy to make decisions in the household had poor screening uptake [15]. As a study from Ethiopia also suggests, improving women’s autonomy can result in the improved use of health services [31].

Despite engaging in the early detection programme, and having higher levels of education and awareness, women who participated in the early detection program were noted to be more fearful of the social stigma of attending ‘cancer clinics’. The fear of being diagnosed with cancer and being socially isolated after diagnosis has been documented in Indian literature on screening for breast cancers. A qualitative study from South India, quoted women’s responses, “[I’m] scared, the family members may not mingle with us casually. If the society comes to know that is all, [I] fear [that], they will isolate us”, or “If they say, we have the disease after the screening, our life is gone after that, the life is over, that kind of negative thoughts [prevent going]” [7]. This throws light on the pervasive fear of cancer diagnosis.

Participants demonstrated more fear than non-participants, especially of losing their entire breast if cancer were to be detected. This could perhaps be attributed to the fact that participants attended awareness talks where they learned about mastectomies and surgical management of the disease. However, it is important to note here that participant women overcame that stigma and fear and presented themselves for examination. Information providers hence must take care to adopt a comprehensive approach to awareness building, emphasising early operative benefits, availability of breast conservation surgery and addressing concerns of patients when they present themselves for consultations. A comprehensive approach aided by empathic communication skills helps prevent fear and foster trust. Yet, further interviews of these participant women to understand mechanisms of coping with fear and stigma to present themselves for screening would be deeply insightful.

The defining parameter of the participant group remained that they had trust in their healthcare providers’ ability to treat them, if they were to be diagnosed with cancer. It is widely recognised that trust in physicians positively influences health-seeking behaviours [32]. Further, Flugelman et al. [33] describe that trust in physicians is the most important factor in reducing patient anxiety prior to an intervention. Another study by Gupta et al. concluded that trust in healthcare providers remained the most important determinant for cancer screening participation, after the already acknowledged barriers of access and affordability [34]. In our study too, despite describing feelings of fear and stigma initially, participants ultimately trusted that their doctors would manage their BC adequately, which helped mitigate their fears.

In contrast, the non-participant group had expressed that the healthcare facility might not be able to adequately treat their BC if it were to be diagnosed and that healthcare workers were rude and indifferent. Given that this was a UHC setting and all patients are life-long members, it is possible that these perceptions among non-participants arose from their previous experiences at other outpatient departments or clinics prior to the study. Hence, they likely did not visit the breast clinics when they were invited to participate. The participants of the program on the other hand perhaps felt more cared for, when they responded to the invites by presenting themselves for breast examination. When they presented, receiving detailed information about cancer and careful handling of their questions and fears probably made them feel confident and reassured about the treating team. The clinics dedicated to breast examination also may have provided a less crowded environment, and the non-rushed, sensitive approach of the caregivers may have further contributed to their trust and confidence in the program. Haworth, Margalit [35] note that South Asian women value healthcare professionals who understand and respect values of personal modesty and shyness. In their study, patients discussed ‘feelings of exposure’ as barriers to attending cervical cancer screening. Trust building and delivering information in a culturally sensitive manner hence becomes a priority to encourage health-seeking behaviours from the patient’s perspective. By improving soft skills of the screening and treating teams, to focus on patient-centric communication and careful redressal of their concerns, patients would be encouraged to trust the system: in order to ensure that the UHC system is utilised to its full potential, trust-building in the context of effective, sensitive communication is paramount. Future work could focus on the impact of communication skills training for physicians and other healthcare professionals in building perceived trust among their patients.

This study has a unique strength in studying a population covered by UHC. As such, this paper was able to analyse socio-cultural barriers outside of other commonly described barriers such as affordability and accessibility. This is further emphasised by our study’s findings that neither participants nor non-participants believed distance to or accessibility of the clinics to be prohibitive to their attendance, making a strong case for UHC. This is in stark contrast to most other publications on the subject. Pal et al. [36] noted financial constraints and infrastructure deficiencies along with low awareness, as significant barriers to seeking breast health services in India.

This study also has certain limitations. It is crucial to note that this study was based in Mumbai, a metropolitan city in India, where access to transport is markedly better than in most rural settings. Hence the generalizability of our findings to rural populations may be limited. A lack of open-ended questions in the methodology also limited the scope of the discussions in this paper. In-depth interviews enabling qualitative analysis would reveal a far more nuanced understanding of socio-cultural barriers, and hence are needed going forward. Future work could also benefit from a more targeted approach and explore the barriers to the uptake of early detection programmes in first-degree relatives of patients with breast cancer, who have been identified as a high-risk cohort [37].

## Conclusions

Higher education, trust, and nuclear families were protective factors and thus facilitators to engaging in the early detection programme. The major socio-cultural barriers identified in the study were joint family setups, lower education and awareness, and a lack of trust in healthcare professionals.

Identifying these socio-cultural barriers to seeking breast health services is essential in order to formulate context-specific solutions to mitigate them. Future work is needed to study these socio-cultural barriers more in-depth. Our study has identified certain implications for changing practice: namely greater trust and awareness building. “Inter-sectorial co-ordination” has previously proven valuable in mobilising different stakeholders for organised cancer detection in low-resource settings [38]. In addition, the engagement of social organisations and communication skills training for healthcare workers will also be essential in building trust among the public. Such multi-pronged approaches could help tackle the vast pool of socio-cultural barriers that exist today, from individual awareness to systemic faults.

### Supplementary Information

**Additional file 1.**

**Additional file 2: Supplementary Table 1.** Regression analysis of factors associated with breast cancer screening.

## Data Availability

The dataset used/analysed in the study is available with the authors AG or PP and can be shared on request for specific research purposes.

## Abbreviations

BC: Breast Cancer
CBE: Clinical Breast Examination
IARC: International Agency for Research in Cancer
HICs: High-Income Countries
LMICs: Low- and Middle-Income Countries
NP: Non-participants
P: Participants
PHCs: Primary Healthcare Centres
UHC: Universal Health Coverage
UIDs: Unique Identification Numbers
WHO: World Health Organization
