# Supplementary material for: Recognising socio-cultural barriers while seeking early detection services for breast cancer: a study from a Universal Health Coverage setting in India
Source: BMC Cancer. 2023 Sep 19;23:881. doi: 10.1186/s12885-023-11359-3 (PMC10507865; doi:10.1186/s12885-023-11359-3)
Supplement: Supplementary file 1 — Additional file 1. [file 12885_2023_11359_MOESM1_ESM.pdf]

Study Sl No

Barriers to Breast Health Seeking and Screening Uptake in an Urban Community in Mumbai, India

(√ Tick wherever appropriate)

Name

CHSS No. /

DOB *(Only year if not remember)*

Designation

Address

A. Socio Demographic Characteristics

1] Education
- 1. Illiterate
- 2. School
- 3.Graduate
- 4.Post graduate
- 9. Unknown

2] Marital status
- 1.Single
- 2.Divorced
- 3.Widowed
- 4. Married
- 9. Unknown

3] Religion
- 1.Hindu
- 2. Muslim
- 3. Christian
- 4. Other
- 9. Unknown

4] Occupation
- 1.Housewife
- 2. Employed
- 3. Professional
- 9. Unknown

5] Menstrual History
- 1. Premenopausal
- 2. Post menopausal
- 3. Hysterectomy
- 9. Unknown

6] Family history of Breast and related cancers
- 1.Yes
- 2. No
- 9. Unknown

7] Medical history of any chronic illness/cancer
- 1.Yes
- *Specify, if Yes*
- 2. No
- 9. Unknown

8] H/o breast feeding
- 1.Yes
- *No.of month,if Yes*
- 2. No
- 9. Unknown

9] Family type
- 1. Joint
- 2. Nuclear
- 9. Unknown

10] Children Alive
- 1
- 2
- 3
- >3

11] Monthly income

B. Questionnaire for barriers to breast health seeking

B1. Awareness

1] Have you heard about the breast cancer ? 1.Yes 2.No 9.Unknown

2] Whom have you heard about cancer from ?

- 1. Family member/Relatives
- 2. Healthcare professionals
- 3 Friends/Neighbours
- 4. TV/Newspaper
- 5 internet
- 6. Multiple source
- 9. Unknwon

3] Which one or many of the following do you think are symptoms of breast cancer?

	1.Yes	2.No	9.Unknown
1. Change in size of the breast			
2. Blood stained nipple discharge			
3. Lump in the breast			
4. Pain in the breasts			
5. Skin changes over the breast			
6. Lump in the arm pit			

Barriers to Breast Health Seeking and Screening Uptake in an Urban Community in Mumbai, India

(√ Tick wherever appropriate)

4] Which of the following are Risk factors for developing breast Cancer ?	1.Yes	2.No	9.Unknown
a. Family history of breast cancer			
b. Alcohol, smoking and other unhealthy life style factors			
c. Overuse of unsupervised contraceptive pills			
d. Not having children or not breast feeding them			
e. Infection from other person having cancer			
5.1] Are you aware that there is routine breast examination of breast carried out for early detection of cancer at your dispensary / Hosp			
5.2] Have you received our Information brochure on Breast awareness ?			

B2 Social and cultural barriers

	1.Yes	2.No	9.Unknown
1] Do you feel it's necessary to get breast checkup done even in absence of symptoms?			
2] Do you feel it is more important to look after health of family members than your own?			
3] Do you feel your family members or spouse will accompany you or encourage you for routine checkup?			
4] Do you feel you do not have time to go for breast checkup ?			
5] Are you afraid that your neighbors or friends may see you as cancer patient if you go for breast checkup?			

B3Geographical barriers

	1.Yes	2.No	9.Unknown
1] Wheather dispensary and breast examination service located far from your residence?			

B4 Perceived Risk

	1.Yes	2.No	9.Unknown
1] Do you think that once you show a doctor, they will find some problem and you will need further treatment?			
2] Do you feel if detected with cancer you will lose your breast			

B5.Fatalism and spirituality

	1.Yes	2.No	9.Unknown
1] Do you think that breast cancer curable ?			
2] Do you think that breast cancer a sin/ punishment for sin from God			
3] Do you think alternative medicines/ spiritual healing is better than doctor's medication or surgery?			

B6. Healthcare related issues

	1.Yes	2.No	9.Unknown
1] Do you think your doctors and hospital can treat you if you are detected with breast cancer?			
2] Have you ever felt there was lack of privacy at the checkup center?			
3] Was the staff rude or indifferent to your desire to get a routine checkup ?			
