# Supplementary material for: Recognising socio-cultural barriers while seeking early detection services for breast cancer: a study from a Universal Health Coverage setting in India
Source: BMC Cancer. 2023 Sep 19;23:881. doi: 10.1186/s12885-023-11359-3 (PMC10507865; doi:10.1186/s12885-023-11359-3)
Supplement: Supplementary file 2 — Additional file 2: Supplementary Table 1. Regression analysis of factors associated with breast cancer screening. [file 12885_2023_11359_MOESM2_ESM.docx]

**Supplementary Table 1. Regression analysis of factors associated with breast cancer screening**

|  |  | **OR (95% CI)** | **p-value** |
| --- | --- | --- | --- |
| Age Group |  |  |  |
|  | <=50 Yrs | reference |  |
|  | >50 Yrs | 1.417 (0.855 - 2.348) | 0.176 |
| Education |  |  |  |
|  | Illiterate | reference |  |
|  | School | 0.558 (0.26 - 1.198) | 0.134 |
|  | Graduate & above | 0.417 (0.183 - 0.946) | 0.036 |
| Marital status |  |  |  |
|  | Partnered | reference |  |
|  | Non-Partnered | 1.534 (0.639 - 3.686) | 0.339 |
| Occupation |  |  |  |
|  | Working | reference |  |
|  | Non-working | 0.869 (0.532 - 1.42) | 0.575 |
| Menstrual Status |  |  |  |
|  | PreMenopausal | reference |  |
|  | PostMenopausal | 0.654 (0.435 - 0.983) | 0.041 |
| F/h/o Breast and related Cancer |  |  |  |
|  | No | reference |  |
|  | Yes | 0.58 (0.331 - 1.017) | 0.057 |
| Medical History |  |  |  |
|  | No | reference |  |
|  | Yes | 1.962 (1.29 - 2.986) | 0.002 |
| Breast Feeding History |  |  |  |
|  | No | reference |  |
|  | Yes | 0.796 (0.359 - 1.767) | 0.575 |
| Family Type |  |  |  |
|  | Nuclear | reference |  |
|  | Joint | 1.463 (0.893 - 2.397) | 0.131 |
